# ROMK expression remains unaltered in a mouse model of familial hyperkalemic hypertension caused by the CUL3^Δ403‐459^ mutation

**DOI:** 10.14814/phy2.12850

**Published:** 2016-07-04

**Authors:** Meena Murthy, Thimo Kurz, Kevin M. O'Shaughnessy

**Affiliations:** ^1^ Division of Experimental Medicine and ImmunotherapeuticsDepartment of MedicineUniversity of CambridgeCambridgeCB2 2QQUnited Kingdom; ^2^ MRC Protein Phosphorylation and Ubiquitylation UnitCollege of Life SciencesUniversity of DundeeDow Street, Dundee DD15EHScotlandUnited Kingdom

**Keywords:** Cullin‐3, hyperkalemia, proteasome, ROMK, ubiquitin‐ligase complex, WNK kinases

## Abstract

Familial hyperkalemic hypertension (FHHt) is a rare inherited form of salt‐dependent hypertension caused by mutations in proteins that regulate the renal Na^+^‐Cl^‐^ cotransporter NCC. Mutations in four genes have been reported to cause FHHt including CUL3 (Cullin3) that encodes a component of a RING E3 ligase. Cullin‐3 binds to WNK kinase‐bound KLHL3 (the substrate recognition subunit of the ubiquitin ligase complex) to promote ubiquitination and proteasomal degradation of WNK kinases. Deletion of exon 9 from CUL3 (affecting residues 403‐459, CUL3^Δ403‐459^) causes a severe form of FHHt (PHA2E) that is recapitulated closely in a knock‐in mouse model. The loss of functionality of CUL3^Δ403‐459^ and secondary accumulation of WNK kinases causes substantial NCC activation. This accounts for the hypertension in FHHt but the origin of the hyperkalemia is less clear. Hence, we explored the impact of CUL3^Δ403‐459^ on expression of the distal secretory K channel, ROMK, both in vitro and in vivo. We found that expressing wild‐type but not the CUL3^Δ403‐459^ mutant form of CUL3 prevented the suppression of ROMK currents by WNK4 expressed in *Xenopus* oocytes. The mutant CUL3 protein was also unable to affect ROMK‐EGFP protein expression at the surface of mouse M‐1 cortical collecting duct (CCD) cells. The effects of CUL3 on ROMK expression in both oocytes and M‐1 CCD cells was reduced by addition of the neddylation inhibitor, MLN4924. This confirms that neddylation is important for CUL3 activity. Nevertheless, in our knock‐in mouse model expressing CUL3^Δ403‐459^ we could not show any alteration in ROMK expression by either western blotting whole kidney lysates or confocal microscopy of kidney sections. This suggests that the hyperkalemia in our knock‐in mouse and human PHA2E subjects with the CUL3^Δ403‐459^ mutation is not caused by reduced ROMK expression in the distal nephron.

## Introduction

The WNK kinases are a family of serine/threonine kinases that are expressed in the mammalian kidney, and mutations in them can cause Gordon's syndrome ( http://omim.org/entry/145260), also known as pseudohypoaldosteronism type 2 (PHA2) or Familial Hyperkalemic Hypertension (FHHt). These kinases connect the aldosterone signaling pathway to renal Na^+^ and K^+^ transporters by regulating the membrane expression of the NaCl transporter (NCC) and the renal outer‐medullary K^+^ channel KCNJ1 (ROMK). The WNK kinases achieve this by forming a signaling cascade with downstream kinases, SPAK (SPS1‐related proline/alanine‐rich kinase) and OSR1 (oxidative stress–responsive kinase 1), which in turn phosphorylate and activate SLC‐type cation‐chloride cotransporters in the kidney, such as NCC, leading to an increased sodium ion reabsorption (Richardson et al. 2008, 2011; Delpire and Gagnon 2008) and hence hypertension due to the salt retention. Subjects with FHHt also have abnormalities in other electrolyte flux pathways showing typically hyperkalemia and metabolic acidosis, that is only partly driven by NCC activation (McCormick et al. 2014; Boyden et al. 2012; Glover et al. 2014; Osawa et al. 2013; Tsuji et al. 2013).

WNK4 has a master regulator role, regulating both the balance between NaCl reabsorption and K^+^ secretion in the kidney. In FHHt, the kidney constitutively reabsorbs NaCl at the expense of an impaired K^+^ secretory pathway (Golbang et al. 2005; Kahle et al. 2003) causing the hyperkalemic hypertension. WNK1, WNK3 and WNK4 inhibit the ROMK activity directly by regulating its expression at the cell surface. This is achieved by stimulating an endocytosis pathway involving clathrin‐coated vesicles (Cope et al. 2006; Welling 2016). The single channel conductance and open/closed probability of ROMK channels are not affected (Leng et al. 2006). Phosphorylation of WNK4 at Ser1196 by serum/glucocorticoid‐induced kinase 1 (SGK1) also blocks the inhibitory action of WNK4 on ROMK. This effect by SGK1 is diminished by the Src‐family protein tyrosine kinase (SFK), which phosphorylate WNK4 and modulate its inhibitory effect on ROMK (Lin et al. 2015).

More recently, mutations in Kelch‐like3 (KLHL3) and Cullin3 (CUL3), components of an E3 ubiquitin ligase complex, were found to cause FHHt (PHA2D http://omim.org/entry/614495 and PHA2E http://omim.org/entry/614496) (Boyden et al. 2012; Louis‐Dit‐Picard et al. 2012). Although mutations in the WNK kinases, KLHL3 or CUL3 cause the same disease, recent reports (Tsuji et al. 2013; Boyden et al. 2012; Osawa et al. 2013) have shown genotype–phenotype differences. Specifically, CUL3 mutations cause an earlier onset and a more severe form of the disease (PHA2E) in terms of both the hypertension and hyperkalemia. All the PHA2E‐causing mutations in CUL3 reported to date, are heterozygous, transmit as autosomal dominant traits and cause splicing defects that delete the amino acids encoded for by exon 9 of the CUL3 mRNA (residues 403–459) (Boyden et al. 2012; Glover et al. 2014; Grimm et al. 2012; Osawa et al. 2013; Tsuji et al. 2013).

Using a knock‐in mouse model of PHA2E, we have reported recently the molecular basis for the effects of the exon 9‐deleted form of CUL3 (Schumacher et al. 2015). This has also suggested that a vascular component could be contributing to the hypertension in addition to the salt retention caused by NCC activation in the DCT. However, the basis for the hyperkalemia was not explored and this forms the basis for this study. Since ROMK is the principal K^+^ secreting channel in the kidney, we have studied the effect of the CUL3 mutation on the WNK4 regulation of this channel using both in vitro and in vivo approaches.

## Materials and Methods

### Expression in *Xenopus* oocytes

All experimentation with Xenopus frogs was carried out in accordance with welfare guidance from the UK Home Office. *Xenopus laevis* oocytes were harvested and defolliculated, and cRNA synthesized as detailed previously (Golbang et al. 2005). Briefly, 50 ng of ROMK (KCNJ1) cRNA alone was injected or ROMK in combination with respective cRNAs for WNK4, CUL3 wild‐type (WT)/CUL3^Δ403‐459^ and KLHL3 WT as the case may be, were coinjected in a total volume of 50 nL per oocyte (The plasmid constructs were a generous gift from Prof. D Alessi, University of Dundee). The cRNA for each of them was run off from the linearized plasmid construct (WNK4 in pTNT, CUL3 WT and mutant forms in pDNA 3.1 and KLHL3 WT in pTLN vector) using the T7 mMessage mMachine Transcription Kit (Ambion^®^ http://www.thermofisher.com/). After injection, oocytes were incubated in HEPES‐buffered ND96 containing 2 mmol/L sodium pyruvate and 0.1 mg/mL gentamicin at 18°C for 2–3 days before use.

### Two‐electrode voltage clamp recording

The methods used have been reported previously in detail (Murthy et al. 2014; Golbang et al. 2005). Water‐injected oocytes were used as controls throughout. In experiments where MLN4924 ( https://www.caymanchem.com/) a small molecule inhibitor of Cullin3 neddlylation was tested, the oocytes were pretreated to a final concentration of 1 *μ*mol/L and DMSO‐treated oocytes were tested as the vehicle control.

### Cell culture

M‐1 CCD cells (a generous gift from Prof JM Edwardson, Department of Pharmacology, University of Cambridge) were grown in standard DMEM/Ham's F12 media with 2.5 mmol/L l‐glutamine adjusted to contain 15 mmol/L HEPES, 0.5 mmol/L sodium pyruvate and 1.2 g/L sodium bicarbonate supplemented with 0.005 mmol/L dexamethasone ( www.sigmaaldrich.com) and 5% fetal bovine serum at 37°C in 5% CO_2_–95% air atmosphere, and transfected using Lipofectamine 2000 (Invitrogen Technologies, https://www.thermofisher.com/) with ROMK‐EGFP alone, or they were cotransfected with WNK4. 24 h post transfection, the cells expressing the ROMK‐EGFP and WNK4 were further split into 10 cm plates and transfected with various combinations of CUL3‐FLAG WT/exon 9 deleted CUL3‐FLAG and KLHL3‐FLAG WT as the case may be. Cells expressing ROMK‐EGFP with WNK4, CUL3 WT, and KLHL3 WT were treated with 3.5 *μ*mol/L MLN4924 for 12 h.

### Biotinylation

Transfected M‐1 CCD cells in 10 cm plates were biotinylated following a previously published method (Hardege et al. 2015). Briefly, M‐1 CCD cells in 10 cm plates were washed once in NES and placed on ice. Cells were incubated in 0.2 mg/mL NHS‐ biotin ( www.thermofisher.com) for 45 min on ice before quenching with Tris buffer (25 mmol/L Tris‐HCl; 150 mmol/L NaCl; 10 mmol/L EDTA, pH 7.4). Cells were removed from the plate and centrifuged at 200 *g* for 5 min at 4°C. Supernatant was discarded and cells incubated in solubilization buffer (25 mmol/L Tris‐HCl; 150 mmol/L NaCl; 10 mmol/L EDTA; 1% Triton X‐100, pH7.4) with protease inhibitors ( www.roche.co.uk) at 4°C with agitation for 1 h. The resulting lysate was cleared by centrifugation at 18,000 *g* rpm 4^°^C for 1 h. 30 *μ*L of streptavidin beads ( www.thermofisher.com) per sample was washed once in wash buffer (25 mmol/L Tris‐HCl; 150 mmol/L NaCl; 10 mmol/L EDTA; 1% Triton X‐100, pH 7.4), centrifuged at 1800 *g* for 5 min at 4°C and supernatant removed. The supernatant was added to streptavidin beads and incubated with agitation for 2 h at 4°C. Beads were then pelleted at 18,000 *g* for 5 min at 4°C and washed three times in wash buffer before resuspending the pellet in sample buffer ( www.lifetechnologies.com).

### Western blotting

Cells were harvested 48 h post plasmid transfection, and they were lysed in 400 *μ*L RIPA lysis buffer supplemented with protease inhibitors, phosphatase inhibitors, and 1 mmol/L DTT. After protein quantification by BCA method, 15 *μ*g equivalent total protein lysate was loaded on to 3–8% Tris‐acetate Nupage gel and transferred to nitroceullulose membranes. When studying ROMK levels in transfected cells, streptavidin beads bound to biotinylated protein were resuspend in sample buffer before loading on gels. Membranes were Ponceau stained to check for transfer efficiency and loading, and blocked with 5% milk for 1 h and incubated with primary antibody in 5% (w/v) skimmed milk in TBS‐T with the indicated primary antibodies: 1 *μ*g/mL WNK4‐total antibody (residues 1221–1243 of human WNK4, S064B) (Division of Signal Transduction Therapy Unit, University of Dundee) or 2 *μ*g/mL rabbit anti‐KCNJ1 antibody which is C‐terminal directed, (Atlas antibodies) and Living Colors monoclonal Antibody (JL‐8) (Clontech) overnight at 4°C. The blots were then washed three times with TBS‐T and incubated for 1 h at room temperature with secondary HRP‐conjugated antibodies diluted 10,000‐fold in 5% (w/v) skimmed milk in TBS‐T. After repeating the washing steps, the signal was detected with enhanced chemiluminescence reagent.

### Immunostaining

Mouse kidney sections on glass slides were prepared for immunostaining according to a previously published protocol (Schumacher et al. 2015). They were then blocked for 2 h at room temperature with 2% (v/v) donkey serum in 0.05% (v/v) Triton X‐100–PBS. Primary antibodies were incubated overnight for 20 h at 4°C at the following concentrations diluted in 1% (v/v) donkey serum in 0.05% (v/v) Triton X‐100–PBS: 2 *μ*g/mL for ROMK ( https://atlasantibodies.com/), 2 *μ*g/mL for total NKCC2 (Division of Signal Transduction Therapy Unit, University of Dundee), and 0.2 *μ*g/mL for Aquaporin2 (AQP2 antibody, Santa Cruz Biotechnology). Two negative controls were processed, one where the primary antibody was absent whereas the secondary antibody was added, and vice‐versa for the second negative control. The former served as a control for autofluorescence due to the secondary antibody. Slides were then washed for 15 min in 0.05% (v/v) Triton X‐100–PBS and incubated in secondary antibody for 2 h in a humid environment. Pre‐absorbed donkey IgG‐conjugated Alexa Fluor 568 and 633 secondary antibodies ( https://www.thermofisher.com/and http://www.abcam.com/) were used at 1:200 diluted in 1% (v/v) donkey serum in 0.05% (v/v) Triton X‐100–PBS for immunofluorescent labeling. Slides were washed as mentioned earlier and mounted using vectashield with DAPI (Vector labs).

### Image acquisition and processing

Immunofluorescent images were acquired on the Leica Sp5 ultrahigh speed inverted confocal microscope with 488‐, 543‐, 633‐nm laser lines mounted on an upright Leica DM RXA fluorescent microscope using an HC PL FLUOTAR 20X/0.5NA objective. Acquisition parameters were: 12‐bit, 1024 × 1024 pixels, 2 × digital zoom, 400 Hz scan speed, sequential (by line) channel imaging and 10 slice z‐stack of 5 *μ*m. In FIJI image analysis software ( http://fiji.sc/), fluorescent z‐stacks underwent background subtraction (1000px radius rolling ball, no smoothing) and average intensity z‐projection. Brightness and contrast were adjusted by linear histogram stretching only to enhance visibility. Comparison kidney images for the CUL3^Δ403‐459/+^ and WT CUL3^+/+^ mouse were processed together using identical image settings.

### Immunoblotting of mouse kidney lysates

All animal studies and breeding was approved by the University of Dundee ethical committee and performed under a UK Home Office approved project license. Snap frozen kidneys from CUL3^+/+^ and CUL3^Δ403‐459^ mice were sliced into quarters, and one of the quarters was homogenized using a TissueLyser LT ( https://www.qiagen.com/#85600) and protein lysates were extracted in RIPA lysis buffers containing protease and phosphatase inhibitors ( https://www.roche.co.uk/ #11836170001 & #04906845001). All steps were carried out at 4°C. Protein concentrations were determined with the Pierce^TM^ BCA protein assay ( https://www.thermofisher.com/#23225). 10 *μ*g equivalent protein lysates were immunoblotted with the rabbit anti‐KCNJ1 antibody ( https://atlasantibodies.com/) at 2 *μ*g/mL concentration in 5% (w/v) skimmed milk in TBS‐T. Secondary antibodies (donkey anti rabbit 800CW (926–32213 at 1:5000 dilution; www.licor.com) and goat anti mouse Alexa680 (A‐21058 at 1:5000 dilution; www. lifetechnologies.com) were incubated in TBS‐Tween for 1 h at room temperature in the dark, then washed 6X in TBS‐Tween. Membranes were imaged and integrated intensity values quantified (bands were normalized against *β*‐actin) using the LiCor odessey system ( www.licor.com) or using IMAGE J software ( http://imagej.net).

### Statistical analysis

All data are shown as mean ± SEM unless indicated otherwise. Significance was determined using unpaired t test or one‐way ANOVA followed by post hoc testing as appropriate. *P* < 0.05 was considered significant. The stats package in Prism4 ( http://www.graphpad.com/scientific-software/prism/) was used. In the plotting of the I–V plots with this package the points are simply connected point‐to‐point and not fitted to any curve.

## Results

### Wild‐type CUL3 and KLHL3 abrogate WNK4 inhibition of ROMK currents

**Figure 1:**
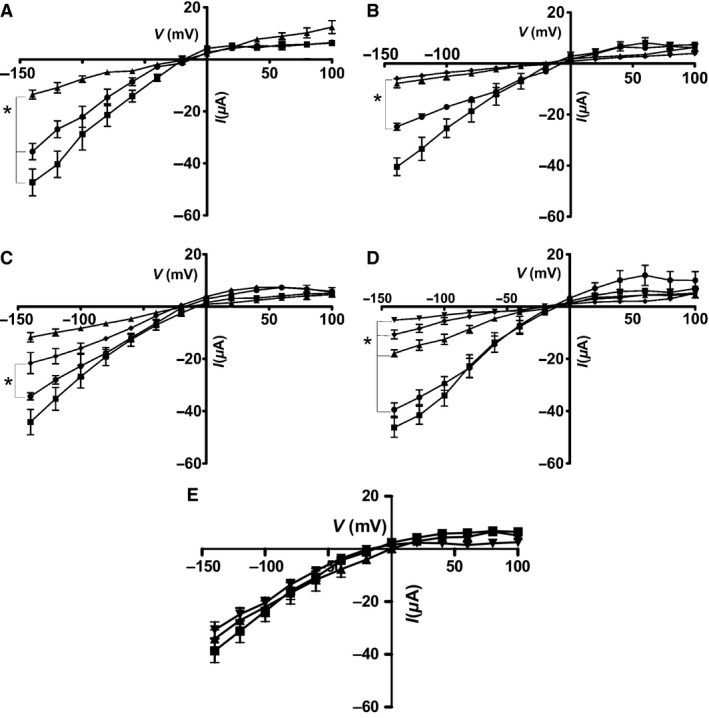
(A) The current–voltage (I–V) plot for voltage‐clamped *Xenopus* oocytes expressing Ba‐sensitive currents. ROMK expressed either alone (■), or ROMK with: WNK4 (▲) or WNK4 +  KLHL3 WT (●). Mean ± sem (*n* = 6), *P <* *1E^−6^ vs ROMK + WNK4. (B) The current–voltage (I–V) plot for voltage‐clamped *Xenopus* oocytes expressing Ba‐sensitive currents. ROMK expressed either alone (■), or ROMK with: WNK4 (▲) WNK4 +  CUL3 WT (●), WNK4 +  CUL3^Δ403‐459^ (♦). Mean ± sem (*n* = 6), *P <* *1E^−6^ versus CUL3. (C) The current–voltage (I–V) plot for voltage‐clamped *Xenopus* oocytes (expressing Ba‐sensitive currents either ROMK alone (■), or ROMK with: WNK4 (▲), WNK4 +  CUL3 WT + KLHL3 WT(●), WNK4 + CUL3 WT + KLHL3 WT + 1 *μ*mol/L MLN 4924 (♦). Mean ± sem (*n* = 6), *P <* *1E^−6^ versus absence of MLN 4924. (D) The current–voltage (I–V) plot for voltage‐clamped *Xenopus* oocytes expressing Ba‐sensitive currents. ROMK expressed either alone (■), or ROMK with: WNK4 (♦), WNK4 +  CUL3 WT (●), WNK4 +  CUL3^Δ403‐459^ (▲) or WNK4 +  CUL3 WT + CUL3^Δ403‐459^ (▼). Mean ± sem (*n* = 6), *P <* *1E^−6^ versus CUL3 alone. (E) The current–voltage (I–V) plot for voltage‐clamped *Xenopus* oocytes expressing Ba‐sensitive currents. ROMK expressed either alone (■), or with CUL3 WT + KLHL3 WT (▲),or CUL3^Δ403‐459^ + KLHL3 WT (▼). Mean ± sem (*n* = 5), No significant differences observed between the three different treatments.

To explore the effect of CUL3 and KLHL3 on WNK4 regulation of ROMK expression, we coexpressed WNK4 and ROMK in *Xenopus* oocytes. In the absence of coexpressed CUL3 or KLHL3, WNK4 produced the expected reduction in ROMK current, whereas KLHL3 WT significantly reduced the WNK4 inhibition of ROMK currents (Fig. 1A). CUL3 or KLHL3 had no effect on ROMK currents in the absence of WNK4 (Fig. 1E). We found that CUL3 wild‐type (WT) completely abolished the effect of WNK4 on the inward component of ROMK currents, but the CUL3^Δ403‐459^ mutant was ineffective (Fig. 1B). The combination of CUL3 and KLHL3 WT had a similar effect on the currents as the individual WT proteins, and pre‐treatment of the oocytes with MLN4924 (Fig. 1C), partially restored the WNK4 inhibition of ROMK. MLN4924 is a small molecule inhibitor of NEDD8‐activating enzyme, (NAE), that inactivates Cullin‐RING E3 ubiquitin Ligases (CRLs) by blocking their NAE‐dependent neddylation. Of note, the effect of the CUL3^Δ403‐459^ mutant was more pronounced than WT CUL3 alone when they were coexpressed with ROMK and WNK4, leading to a further decrease in ROMK currents (Fig. 1D). This suggests that the CUL3^Δ403‐459^ mutant form of CUL3 functions as a dominant‐negative on CUL3 WT.

### WNK4 is a substrate for the CUL3‐KLHL3 complex in vitro

**Figure 2:**
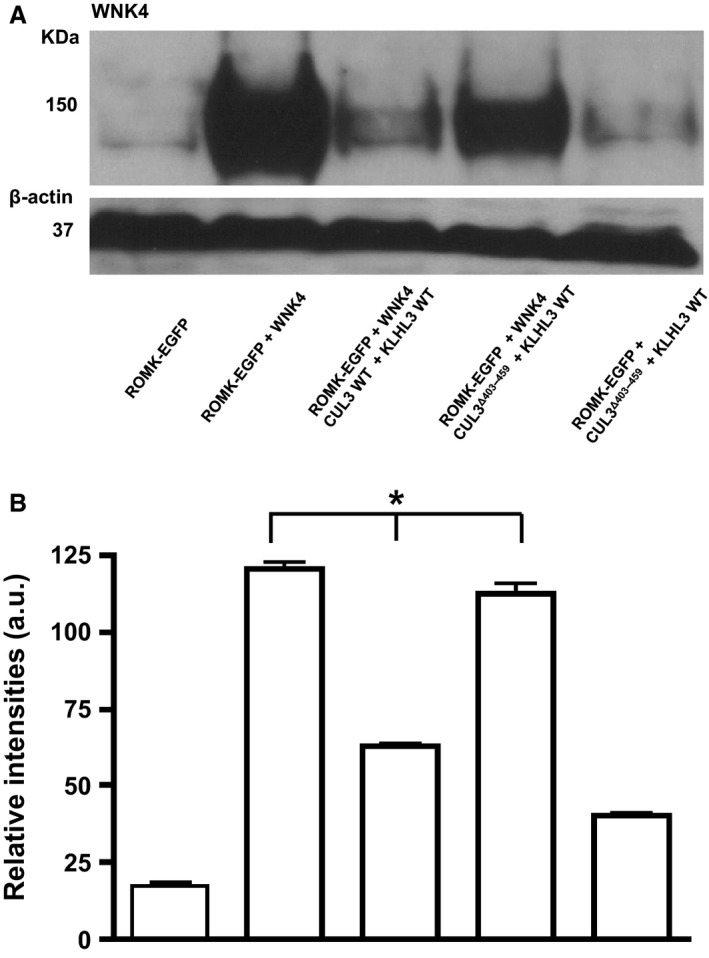
(A) Effect of CUL3 on the WNK4 protein levels in the mouse M‐1 CCD cell line. Total protein lysates were blotted from M‐1 CCD cells transfected with ROMK alone (far left) or ROMK with the constructs indicated. Lane 5 (far right) is a control where WNK4 has not been transfected into the cells. Anti‐WNK4 antibody (residues 1221–1243 of human WNK4, S064B) detected a 150 KDa WNK4 band. *β*‐actin was the loading control. This is representative of Western blots replicated three times with cell lysates from three different passage numbers. (B) Quantification of Western blots showing WNK4 levels normalized to its *β*‐actin loading control. Error bars represent mean ± sem of Western blots (*n* = 3). *P <* *0.0001 by one‐way ANOVA.

To study the effect of the CUL3‐KLHL3 complex on WNK4 protein abundance, ROMK‐EGFP, WNK4, and KLHL3 WT were coexpressed with CUL3 WT or the CUL3^Δ403‐459^ mutant form, respectively, in a mouse cortical cell line (M‐1 CCD). Low endogenous WNK4 levels were detectable in transfected M‐1 cells but were markedly increased by transfection (Fig. 2). Coexpression of WT CUL3 with KLHL3 substantially reduced the level of immunoblottable WNK4, but the CUL3^Δ403‐459^ mutant was much less effective (Fig. 2).

**Figure 3:**
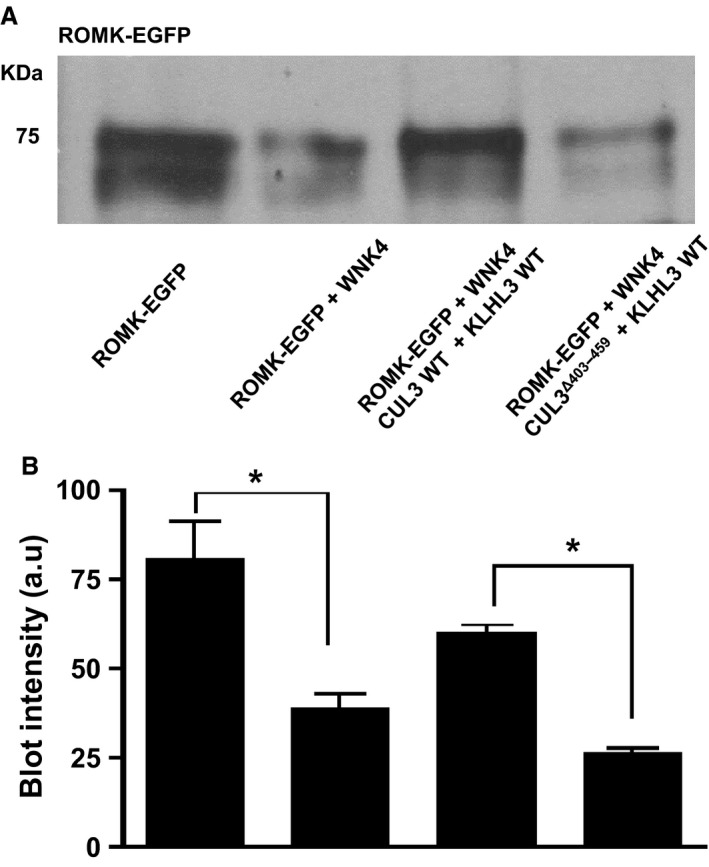
(A) Effect of CUL3 on the biotinylated ROMK‐EGFP levels in the M‐1 CCD cell line. Total protein lysates were blotted from M‐1 CCD cells transfected with ROMK‐EGFP alone or ROMK‐EGFP with constructs indicated. Anti‐KCNJ1 antibody detected a band at around 75 KDa for ROMK‐EGFP. This is representative Western blots replicated 3 times with cell lysates from three different passage numbers. (B) Quantification of Western blots showing ROMK levels in biotinylated cell lysates. Error bars represent mean ± sem of Western blots (*n* = 3). *P <* *0.05.

**Figure 4:**
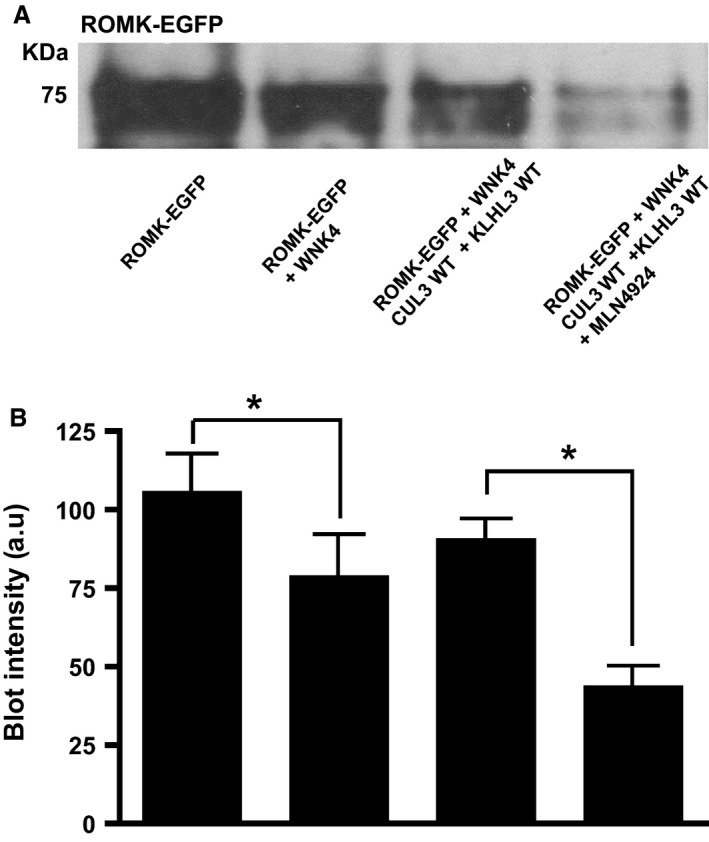
(A) Effect of CUL3 on the biotinylated ROMK‐EGFP levels in the presence of WNK4 in a mouse cortical collecting duct cell line (M1 CCD). (A) Total protein lysates were blotted from M‐1 cells transfected with ROMK‐EGFP alone or ROMK‐EGFP with the constructs indicated. The last combination was also treated with 3.5 *μ*mol/L MLN4924. Anti‐KCNJ1 antibody detected a band at around 75 KDa for ROMK‐EGFP. This is representative of Western blots replicated with three different passage numbers. (B) Quantification of Western blot showing ROMK levels in biotinylated cell lysates. Error bars represent mean ± sem of three Western blots. *P <* *0.05 by one‐way ANOVA.

The M‐1 cells were then biotinylated to measure ROMK channel protein expressed at cell surface. Western blotting with the anti‐KCNJ1 antibody showed that the surface expression of ROMK moved in parallel with the variations in WNK4 levels in the transfected M‐1 cells (Fig. 3). WNK4 significantly reduced the ROMK‐EGFP levels as compared to ROMK‐EGFP alone. In cells expressing CUL3 WT, the ROMK‐EGFP levels were restored whereas this salvage effect of CUL3 was greatly reduced in CUL3^Δ403‐459^ mutant expressing cells, emphasizing the contrasting effects of the WT and CUL3^Δ403‐459^ mutant form of CUL3 on WNK4 levels. To further investigate the effect of CUL3 on WNK4 levels in M1 CCD cells, the CUL3 WT transfected cells were treated with the NEDD8‐activating enzyme inhibitor, MLN4924 to block neddylation and activation of the CUL3‐ligase complex. In keeping with the effect of this inhibitor on ROMK currents in oocytes (Fig. 1C), ROMK‐EGFP levels were markedly reduced in the MLN4924‐treated M‐1 cells (Fig. 4), showing the importance of CUL3 Neddylation for its effect on WNK4 degradation and hence ROMK‐EGFP expression.

### ROMK expression *in vivo* in the CUL3^Δ403‐459/+^ mouse

**Figure 5:**
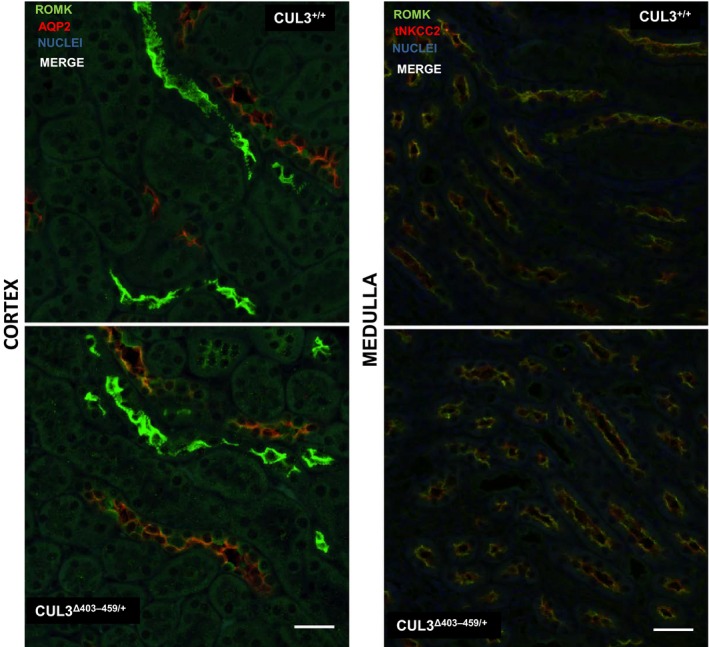
Immunostaining of ROMK (green) and AQP2 (red) in the cortex and apical immunostaining of ROMK (green) and total NKCC2 (red) in the medulla of CUL3^Δ403‐459/+^ kidney versus littermate controls CUL3^+/+^. These are representative pseudocolored average intensity z‐projections of immunofluorescent‐stained kidney sections (4 per genotype) and four independent immunostaining experiments, showing the distribution of total protein. Colocalization is in orange. Scale bar = 30 *μ*m.

**Figure 6:**
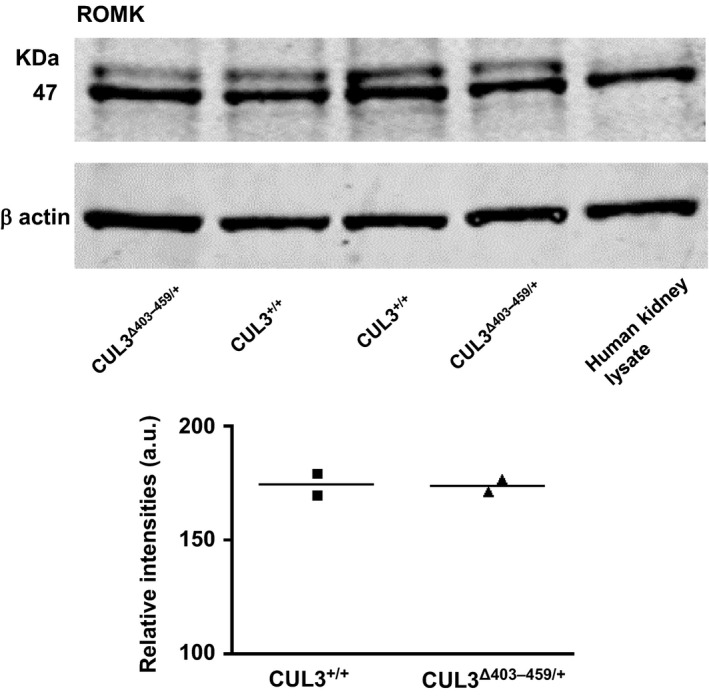
ROMK levels in the CUL3^Δ403‐459/+^ versus CUL3^+/+^ mouse kidney. Total protein from whole mouse kidney lysates immunoblotted with anti‐KCNJ1 antibody detected a band at ~47 KDa in both the mouse and human kidney lysates (as positive control). *β* actin was the loading control. The individual densitometry of each lane is shown (the bar is the average).

To establish if expression of the CUL3^Δ403‐459^ mutant form of CUL3 was able to alter expression of ROMK in vivo, we studied our recently characterized knock‐ in CUL3^Δ403‐459/+^ mouse. This mouse recapitulates closely the human PHA2E phenotype including its hyperkalemia. Using confocal immunofluorescent staining we identified ROMK in the cortical collecting ducts (CCD), where it co‐localized with AQP2, connecting tubules (CNT), and the thick ascending limb (TAL) in the medulla of these mice (Fig. 5). ROMK also colocalized with NKCC2 in the TAL as expected. However, there were no obvious differences in either the intensity or distribution of the staining in CUL3^Δ403‐459/+^ mouse versus the WT CUL3^+/+^ littermate controls. Immunoblotting whole kidney lysates also failed to identify any differences in ROMK abundance in the CUL3^Δ403‐459/+^ in keeping with the results of the immunohistochemical staining (Fig. 6).

## Discussion

We recently reported a knock‐in mouse model in which heterozygous expression of CUL3^Δ403‐459^ recapitulates closely the human PHA2E phenotype (Schumacher et al. 2015). Molecular modeling further suggested that the deletion of exon 9 (residues 403–459) increased structural flexibility of the CUL3 protein that affected its scaffolding function. Specifically, mutant CUL3 binds to the RBX1 protein to form a ubiquitin E3 ligase, but the complex cannot effectively degrade KLHL3‐bound substrates such as WNK4 (Schumacher et al. 2015). Instead, CUL3^Δ403‐459^ auto‐ubiquitylates and loses its interaction with two important Cullin regulators: the COP9‐Signalosome and CAND1. The defective ubiquitylation of WNKs led to substantial accumulation in the kidney of WNK4 and its downstream components, SPAK and NCC (both their total and phosphorylated forms). This scenario provided an explanation for the hypertension in the mice, but it was unclear whether it also explained the hyperkalemia.

Our previous work on WNK kinase regulation of ROMK showed that WNK1 reduces surface expression of ROMK in *Xenopus* oocytes by stimulating internalization of the channel through a pathway involving clathrin‐coated vesicles (Cope et al. 2006). An identical mechanism is reported to operate for WNK4 at least in cultured mammalian cells (Kahle et al. 2003). Hence, our working hypothesis was that WNK4 accumulation in the kidney of the CUL3^Δ403‐459^ mouse could be sufficient to cause substantial reduction of ROMK expression in the distal nephron. This loss of ROMK from the luminal membrane would in turn provide an explanation for the hyperkalemia seen in our knock‐in mouse.

The studies reported here in *Xenopus* oocytes confirms that overexpression of the WNK4 is able to substantially reduce ROMK currents. We further show that this effect is abolished by coexpression of CUL3 protein but not by expressing the CUL3^Δ403‐459^ mutant. This presumably reflects the ability of CUL3 (but not the mutant) to form an active Cullin‐RING‐ligase with *Xenopus* paralogs of Kelch‐like3, which ubiquitylates the overexpressed WNK4 causing its endosomal degradation. This is supported by the effect of MLN4924, an inhibitor of CUL3 Neddylation. In the presence of MLN4924, the ability of CUL3 WT to reverse the effect of WNK4 on ROMK current was lost, showing that ubiquitylation through an active RING‐ligase is indeed necessary for the action of CUL3 in these experiments.

Coexpressing CUL3 WT and the CUL3^Δ403‐459^ mutant in the oocytes did not restore the ROMK currents suggesting that the mutant behaves as a dominant negative in this system. This contrasts with our earlier published findings in mammalian HEK cells, where we speculated that the CUL3^Δ403‐459^ mutant may cause haploinsufficiency because of its extensive removal by auto‐degradation. However, our observations here are supported by other recent studies which have also suggested that the CUL3^Δ403‐459^ mutant behaves as a dominant negative being more effective than the CUL3 WT in reducing the abundance of the WNK4 adaptor, KLHL3 (Araki et al. 2015; Ibeawuchi et al. 2015; McCormick et al. 2014). Hence, the mutant might effectively increase WNK4 abundance by degrading the endogenous paralogs of KLHL3 in *Xenopus* oocytes.

Our findings in the oocytes were replicated in the mouse M‐1 CCD cells where we have blotted WNK4 to confirm its predicted accumulation in the presence of the CUL3^Δ403‐459^ mutant. An increase in WNK4 levels in the presence of CUL3 mutant led to a corresponding decrease in the abundance of biotinylated ROMK in M‐1 cells. This confirms that the accumulated WNK4 is able to alter the abundance of ROMK at the cell surface of CCD cells, and vice‐versa. To see if the mechanism actually operates in vivo we looked at ROMK expression in the kidneys of our CUL3^Δ403‐459/+^ mice and compared them with CUL3^+/+^ littermates. However, it was obvious from confocal images of immunostained kidney sections that that there were no convincing differences in ROMK levels in the CNT, TAL or the CCD of the two genotypes. Furthermore, there were no differences detectable by immunoblotting whole kidney lysates from the two genotypes. It is possible, however, that significant differences in vivo ROMK currents have gone undetected. This is technically challenging to perform but could be measured by ex vivo patch clamping of CD tubules. Alternatively differences may be uncovered by altering dietary K or measuring the saluretic responses amiloride challenge(Hadchouel et al. 2010).

Clearly, the knock‐in mouse does not recapitulate in vivo the reduced ROMK expression seen in vitro despite high WNK4 levels in both systems. In fact in the kidney of the CUL3^Δ403‐459/+^ mice the excess WNKs (and downstream signaling components) accumulate to form discrete puncta within the cytosol (Schumacher et al. 2015). Nevertheless, mouse models of FHHt have generally not reported on ROMK expression in vivo to provide a comparison for our observations. One exception is a mouse showing kidney‐specific knock‐out of WNK1 that was reported to have modified ROMK staining in the DCT and CNT (Hadchouel et al. 2010). However, this was based only on a visual impression and the authors commented that there was marked variability between animals and tubules in the intensity of apical staining. The same group also reported reduced expression of ROMK of mice overexpressing WNK1‐LS in the DCT. Again the data relied on a visual comparison of confocal images (Vidal‐Petiot et al. 2013). No attempt was made to follow this visual impression with quantification such as western blotting. The mouse could, of course, become hyperkalemic secondary to the high levels of NCC activity in the DCT reducing electrogenic Na transport in the CCD. This would reduce the potential difference needed to drive K secretion by ROMK channels regardless of their expression level. It is also possible that other mechanisms operate to reduce K secretion. For example, recent studies have shown that BK channels are involved in flow‐induced K^+^ secretion in the distal nephron and are regulated by WNK4 in an in vitro system (Wang et al. 2013). This study indicated that WNK4 inhibits BK channel activity partly by degradation of the channel protein through an ubiquitin‐dependent pathway. Hence, WNK4 accumulation could directly suppress BK expression and this forms the subject of future investigations.
